# The Use of Conical Powered Burrs in Surgery of the Bony Nasal Pyramid

**DOI:** 10.3390/jcm15114335

**Published:** 2026-06-03

**Authors:** Marcin Jadczak, Paweł Rozbicki, Wojciech Kurzyński, Dariusz Jurkiewicz, Sandra Krzywdzińska

**Affiliations:** Department of Otolaryngology with Division of Cranio-Maxillo-Facial Surgery, Military Institute of Medicine—National Research Institute, 04-141 Warsaw, Poland; prozbicki@wim.mil.pl (P.R.); wkurzynski@wim.mil.pl (W.K.); djurkiewicz@wim.mil.pl (D.J.)

**Keywords:** rhinoplasty, rhinoseptoplasty, powered instrumentation, conical burrs, cylindrical burrs, osteotomy, nasal dorsum

## Abstract

**Background**: Rhinoseptoplasty is regarded by many surgeons as one of the most demanding procedures in aesthetic surgery. The introduction of new surgical techniques and instruments aims to improve treatment outcomes, increase procedural safety and reproducibility, reduce soft-tissue trauma and shorten operative time. The development of powered instrumentation has contributed substantially to progress in nasal surgery by supporting these goals. The aim of this study was to compare the aesthetic and functional outcomes of rhinoseptoplasty performed with powered instrumentation (PI) and with conventional methods (CR). **Methods**: The study included 106 patients (78 women, 28 men) aged 18–65 years (mean 34 ± 9.8) who were qualified for surgery. CR were used in 62 (58.5%), while PI were used in 44 (41.5%). Patients completed the Rhinoplasty Outcome Evaluation (ROE) and the Standardized Cosmesis and Health Nasal Outcomes Survey (SCHNOS) questionnaires before surgery and 6 months postoperatively. The data were statistically analyzed. **Results**: Both groups demonstrated statistically significant postoperative improvement in all assessed questionnaire outcomes (*p* < 0.0001). ROE scores increased, whereas total SCHNOS, SCHNOS-O, and SCHNOS-C scores decreased. Effect-size analysis indicated a stronger therapeutic effect in the PI group than in the CR group: ROE Cohen’s d, 3.48 vs. 3.07; total SCHNOS r, 0.99 vs. 0.80; SCHNOS-O r, 0.85 vs. 0.62; and SCHNOS-C r, 0.97 vs. 0.93. No major intraoperative complications were observed. **Conclusions**: Meaningful gains in patient-reported outcomes were observed following rhinoseptoplasty in this cohort. The findings provide a rationale for future studies examining whether powered instrumentation offers measurable advantages in selected patients and surgical contexts.

## 1. Introduction

Plastic surgery of the external nose is considered one of the most challenging procedures in modern aesthetic surgery. Increasing social pressure, the emphasis on a flawless appearance, the ubiquity of social media, and the growing dependence of subjective well-being on appearance encourage patients to seek help in surgical practice. On the one hand, patients are increasingly aware of the course of surgery, the complexity of operative techniques, and the need to find experienced medical personnel. On the other hand, acceptance of operative risk, possible complications, and the potential need for revision surgery is decreasing. These factors compel surgeons to continually seek new operative techniques that improve final outcomes without increasing the risk of complications [1,2].

In recent decades, the introduction of piezoelectric devices and other forms of powered instrumentation has enabled substantial progress in reconstructive and aesthetic surgery. Drills and burrs have long been an integral part of the surgical armamentarium in orthopedics, maxillofacial surgery, and otorhinolaryngology. They have been used primarily to contour osseous structures in the treatment of trauma, degenerative and inflammatory changes, and congenital deformities. These instruments not only accelerate surgical procedures but also provide more refined control, which has led to broader use in orthognathic surgery, genioplasty, otology and paranasal sinus surgery [3,4,5].

During rhinoseptoplasty, the upper one-third of the nose has traditionally been treated with manual instruments such as chisels, rasps, osteotomes, and saws [6]. The introduction of powered instrumentation into clinical practice in the 1990s represented an important step toward improving the predictability, precision, and safety of nasal surgery. The use of drills and burrs reduced the risk of undesirable soft-tissue injury, uncontrolled fractures of nasal bony structures, fragmentation, and instability of the nasal pyramid. Moreover, it created new possibilities for bone contouring without the need for conventional osteotomies and mobilization. It should be emphasized, however, that ball-shaped burrs used at that time carried a certain risk of creating small depressions on the bone surface because of their limited working area. These irregularities could produce contour abnormalities palpable to the patient and, in some cases, clinically visible. This problem was particularly relevant in patients with thin skin overlying the bony nasal pyramid, in whom even minor irregularities may have greater aesthetic importance [7,8,9]. Contemporary drill tips are better adapted to the anatomy and allow more precise bone preparation, thereby reducing the risk of surface irregularities. They therefore appear more accurate than earlier solutions and may, in many cases, represent a favorable alternative to conventional methods [7].

The aim of this study was to evaluate and compare the aesthetic and functional outcomes of rhinoseptoplasty, analyzed using validated questionnaires, in patients operated on with surgical burrs and piezoelectric instruments and in patients in whom osteotomy was performed using conventional methods with chisels/osteotomes and rasps.

## 2. Materials and Methods

The study included 106 patients (78 women and 28 men) aged 18–65 years (mean age 34.0 ± 9.8 years) admitted to the Department of Otolaryngology of the Military Institute of Medicine—National Research Institute for nasal airway obstruction and external nasal deformity, who were subsequently qualified for surgical treatment. Sixty-two patients (58.5%) underwent surgery with conventional instrumentation (CR), and 44 patients (41.5%) underwent surgery with powered instrumentation (PI). All procedures were performed by the same surgical team. Patients were allocated to the CR and PI groups according to the surgical technique applied. Group allocation depended on routine operative conditions, including the availability of surgical equipment at the time of surgery, rather than surgeon preference or anatomical considerations, in order to avoid systematic bias in treatment selection that could affect statistical comparisons. No randomization was performed. The study protocol did not assign patients to a specific intervention or surgical technique, and participation in the study did not influence treatment selection. The study was observational in nature and involved the prospective collection of patient-reported outcome measures. Ethical approval was obtained from the Bioethics Committee of the Military Institute of Medicine—National Research Institute (resolutions KB/39/23 and KB/24/24, dated 11 April 2023 and 7 May 2024).

A detailed medical history was obtained from all patients, and standardized photographic documentation was performed before surgery and at each postoperative follow-up visit. The documentation included frontal, lateral (profile), basal, oblique and dorsal views of the nose (helicopter view). The indications for surgery, the nature of the problem and the expected treatment outcomes were discussed individually with each patient in a manner adapted to the patient’s understanding.

Rhinoseptoplasty procedures were performed under general endotracheal anesthesia using an external approach through an inverted V-shaped transcolumellar incision.

Patients completed questionnaires before and 6 months after surgery. The questionnaires included the Rhinoplasty Outcome Evaluation (ROE) and the Standardized Cosmesis and Health Nasal Outcomes Survey (SCHNOS).

The ROE is a concise, self-administered questionnaire used to measure patient-reported satisfaction after rhinoplasty. It comprises targeted questions that capture both aesthetic and functional dimensions of nasal surgery while also reflecting its broader impact on health-related quality of life [10].

The SCHNOS is a validated instrument designed to assess outcomes of nasal surgery in a multidimensional manner. It integrates items addressing two key domains: cosmetic appearance and nasal obstruction. By evaluating form and function simultaneously, SCHNOS provides a comprehensive and reliable assessment of the patient’s postoperative experience. Its strength lies in combining functional and aesthetic perspectives, allowing clinicians to capture both the degree of nasal obstruction and the patient’s perception of nasal appearance. The questionnaire is divided into two principal domains: the obstruction domain (SCHNOS-O; questions 1–4), which evaluates breathing function and nasal airflow and the cosmesis domain (SCHNOS-C; questions 5–10), which includes decreased mood and self-esteem due to the nose, shape of the nasal tip, straightness of the nose, shape of the nose from the side, how well the nose suits the face and overall symmetry of the nose [1,11].

All patients provided informed consent to participate in the study and for the processing and use of their images for scientific purposes. During the course of the study, 17 patients (11 men and 6 women) were excluded from the final analysis because of incomplete or inaccurately completed ROE and/or SCHNOS questionnaires or failure to attend scheduled follow-up visits. The collected data were analyzed statistically.

The inclusion criteria were age above 18 years, deviation of the nasal pyramid, nasal airway obstruction and absence of a clear expected advantage of one surgical method over the other. The exclusion criteria were age below 18 years, active infection, lack of consent to participate in the study, incomplete or missing preoperative and/or postoperative questionnaires and a situation in which one surgical technique was expected to be clearly superior.

For CR conventional hand osteotomes, chisels and rasps were used. For PI the surgeon used powered instrumentation with cylindrical and conical burrs and an ultrasonic piezoelectric bonecutting system.

In the PI group, osteoplasty was performed using a micromotor-driven powered drill system (ORIGO system with a RAPIDO micromotor and compatible handpieces and burrs, including 4- and 6 mm cylindrical burrs and 4- and 6 mm conical burrs; Bien-Air Surgery SA, Le Noirmont, Switzerland). Osteotomy was performed using a Surgic Touch Piezo Bone Surgery device (Guilin Woodpecker Medical Instrument Co., Ltd., Guilin, China). During osteoplasty, cylindrical and conical burrs were used at rotational speeds below 15,000 rpm, which provided precise work while avoiding overly aggressive removal of bony structures. Sterile physiological saline (0.9% NaCl) was used to irrigate the operative field. Irrigation cooled the operating area, washed away bone dust, and improved tissue visualization during surgery. A retractor with an integrated suction channel was used to evacuate fluid and bone dust.

### Statistical Analysis

After the data obtained before surgery and after 6 months of follow-up were tabulated, the study population was analyzed with respect to sex, age and questionnaire results (ROE, total SCHNOS, SCHNOS items 1–4 and SCHNOS items 5–10). Patients were then divided into subgroups according to the type of operation performed.

In the next stage, the distribution of variables in the subgroups was assessed using the Kolmogorov–Smirnov test. When a normal distribution was confirmed, differences between groups were analyzed using Student’s *t*-test. When statistical significance was obtained, effect size was calculated using Cohen’s d to assess the strength of the observed differences. To quantify the differences between groups, 95% confidence intervals (95% CI) were calculated.

For subgroups in which the distribution deviated from normality, the Mann–Whitney U test was used. When statistically significant differences were found, the rank correlation coefficient (r) was additionally calculated to evaluate effect size.

Statistical analysis was performed using Statistica 7.0 software (StatSoft Inc., Tulsa, OK, USA) and Microsoft Office.

## 3. Results

The analyzed group comprised 106 patients (78 women and 28 men) with a mean age of 34.0 years (±9.8), admitted to the Department of Otolaryngology of the Military Institute of Medicine- National Research Institute for rhinoseptoplasty. The Kolmogorov–Smirnov test confirmed a normal age distribution in the study group (*p* > 0.05). Table 1 presents the questionnaire results for the entire study group.

The entire cohort was then divided according to the type of surgery: conventional instrumentation (CR; n = 62) and powered instrumentation (PI; n = 44). Age and sex distribution were analyzed within each subgroup, and questionnaire results were then verified (Table 2). For all variables except postoperative SCHNOS and postoperative SCHNOS-C, normal distribution was confirmed.

Table 3 presents the characteristics of the CR rhinoseptoplasty subgroup, including age and sex distribution. Normal distribution was observed for preoperative and postoperative ROE, preoperative SCHNOS, and preoperative SCHNOS-O. Normal distribution was not observed for the remaining variables.

After subgroup characteristics had been tabulated, Student’s *t*-test and Cohen’s d were used for comparisons involving normally distributed variables, whereas the Mann–Whitney U test and the r coefficient were used for the remaining comparisons. The results are presented in Table 4.

The analysis demonstrated statistically significant improvement in all assessed groups (*p* < 0.0001), with decreased SCHNOS scores and increased ROE scores. Effect sizes (Cohen’s d and correlation coefficients) and 95% confidence intervals were calculated during post hoc statistical analysis to quantify the magnitude and precision of the observed effects. Observed statistical power was also estimated based on the obtained effect sizes. However, the magnitude and consistency of improvement were greater in the PI group than in the CR group (ROE Cohen’s d: 3.48 vs. 3.07; total SCHNOS r: 0.99 vs. 0.80; SCHNOS-O r: 0.85 vs. 0.62; SCHNOS-C r: 0.97 vs. 0.93). These findings, considered together, indicate a potential advantage of PI over CR.

## 4. Discussion

Every nose is to some extent asymmetrical and forms part of an asymmetrical face. The goal of well-performed rhinoseptoplasty is the most complete possible correction of these disproportions while maintaining a natural and aesthetically balanced final outcome. In the frontal view, an aesthetic nose is characterized by distinct lines separating the dorsum from the lateral nasal walls. These lines originate in the bony portion and smoothly transition into the cartilaginous part. The curved course of the aesthetic lines begins at the medial end of the eyebrows, runs along the nasal dorsum and extends toward the nasal tip. These lines should be symmetrical, gently narrowing in the radix region and then gradually widening toward the nasal tip [6,12].

On frontal photographs, the nasal dorsum appears as a lighter band limited by the aesthetic lines, whereas the lateral walls create subtle shadows running almost vertically from the medial eyebrow region toward the supra-alar area. During surgery, particularly structural rhinoseptoplasty (SR), the original architecture of the dorsum is substantially altered, temporarily disrupting the aesthetic lines. The purpose of rhinoseptoplasty is to restore a harmonious shape of the dorsum and lateral walls of the nasal pyramid by correcting an excessively wide dorsum, asymmetry, convex nasal bones and sharp transitions between the bony and cartilaginous portions [13,14].

Studies indicate that the nasal dorsum is formed largely by cartilaginous components, whereas the lateral wall of the nasal pyramid is formed by the nasal bones and the frontal processes of the maxilla. Although an attractive frontal nasal surface is largely achieved during nasal surgery by reconstructing the middle third with spreader grafts or spreader flaps, a smooth transition between the upper third and the middle third is achieved by appropriate shaping of the nasal bones, which is one of the key elements of rhinoseptoplasty [15,16]. Osteotomy, as a key stage of this procedure, consists of intentional cutting of the nasal bones to allow their subsequent repositioning and to obtain the desired shape of the nasal pyramid. This procedure is performed, among other reasons, to narrow a wide nose, close deformities created after hump removal and correct deviation. At the same time, it is considered the most traumatic and least predictable stage of surgery [17].

The precision of osteotomy has a direct influence on the final outcome, both aesthetically and functionally, with regard to nasal airway patency. An incorrectly directed osteotomy line may result in dorsal irregularities, asymmetry, displacement or instability of bony fragments and excessive narrowing or collapse of the nasal vault, which may secondarily lead to breathing impairment. Conversely, a precise and controlled bone cut enables proper repositioning, preservation of symmetry, and a stable and predictable postoperative result [18,19,20]. Limiting trauma to surrounding tissues is also important. Excessive force or uncontrolled fractures may cause greater swelling, hematoma formation, periorbital ecchymosis and prolonged recovery. Precise osteotomy minimizes the risk of injury to the mucosa, vessels, and nasal support structures, thereby reducing postoperative complaints and accelerating functional recovery.

The choice of the incision site, operative technique, instruments, and force required for osteotomy depends largely on each patient’s anatomy, particularly the thickness and density of the nasal bones, their susceptibility to modeling, and the presence of previous trauma or prior surgery. In patients with thicker and more compact bones, greater force may be required when using conventional instruments such as chisels; however, this is associated with less control over the fracture line. Modern technologies, including burrs and piezoelectric devices, can provide an alternative by allowing precise and controlled cuts and reducing the risk of undesired fractures and soft-tissue injury. The choice of osteotomy technique (internal vs. external, low vs. high, continuous vs. perforating) should also be adapted to the intended aesthetic result and the anatomic conditions, highlighting the importance of surgeon experience and individualization of surgical management [20].

For many years, instruments such as rasps, chisels, and bone saws have been used to shape the bony part of the nasal dorsum. Modification of the shape and size of the nasal bones, the frontal processes of the maxilla, and the nasal processes of the frontal bone is required regardless of the operative concept used. This applies to both structural rhinoplasty (SR) and preservation rhinoplasty (PR). Manual instruments, however, cannot fully guarantee a precise and predictable effect of the surgeon’s maneuvers. An additional challenge during surgery is the final shaping of the nasal bones after osteotomy, because manipulating conventional instruments on mobile bone fragments may be difficult [21,22,23,24].

**Figure 1:**
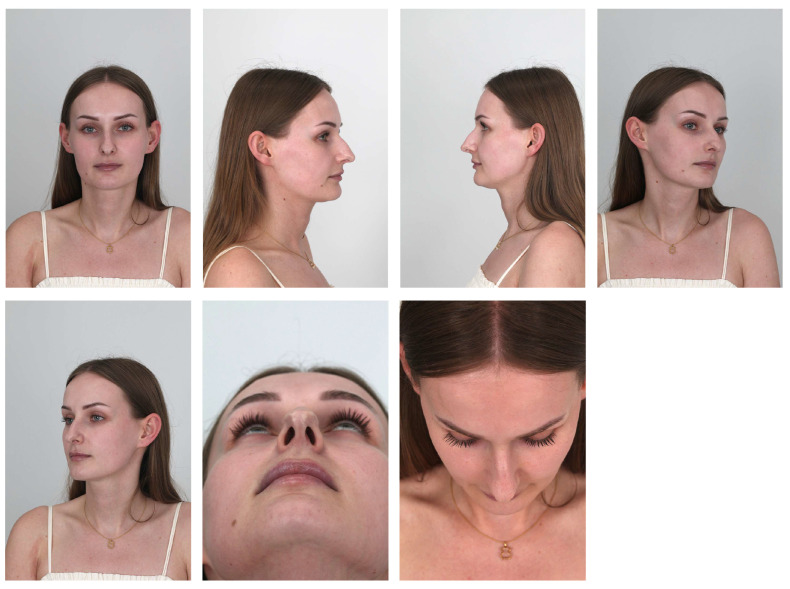
Preoperative photographs of a female patient undergoing rhinoseptoplasty with conventional instrumentation (CR).

**Figure 2:**
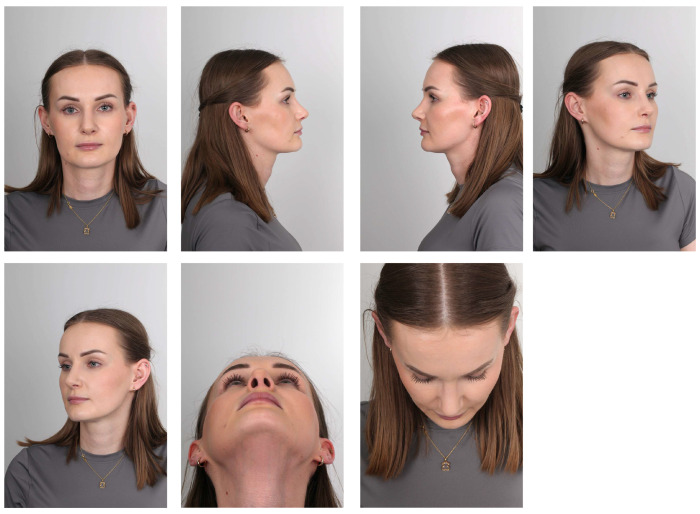
Six-month postoperative photographs of the same female patient after rhinoseptoplasty with conventional instrumentation (CR).

In the present study, postoperative assessment of the 62 patients operated on with CR demonstrated significant improvement in both ROE and SCHNOS scores (Table 4). Compared with the preoperative state, the improvement was statistically significant. These results indicate a clear increase in patient satisfaction with external nasal appearance, subjective nasal patency, and breathing comfort. The observed changes confirm that CR may be an effective surgical approach for patients requiring simultaneous aesthetic and functional correction. Figure 1 shows a patient before surgery performed with CR, whereas Figure 2 shows postoperative photographs taken 6 months after rhinoseptoplasty with hand instrumentation. These results also emphasize the value of standardized assessment tools, such as ROE and SCHNOS, in objectifying treatment outcomes and monitoring postoperative results in rhinoseptoplasty.

At present, powered instrumentation is increasingly used because it may allow more precise and controlled modification of bony structures than traditional hand instruments such as chisels and rasps. Burrs enable delicate and accurate bone contouring and minimize the risk of creating irregular edges or uncontrolled fractures. They make it possible to obtain a smooth transition between the lateral nasal wall and the facial contour, correct bony asymmetries effectively, and harmoniously shape the upper and middle thirds of the nose while maintaining a natural transition toward the upper lateral cartilages. By design, this approach facilitates predictable and aesthetic operative outcomes and is assumed to increase procedural safety [8,25].

In the authors’ cohort, comprising patients operated on with PI (n = 44) and CR (n = 62), postoperative analysis demonstrated statistically significant improvement in all assessed parameters. SCHNOS scores decreased significantly, whereas ROE scores increased, indicating both functional and aesthetic improvement (*p* < 0.0001; Table 4). A greater improvement in the author’s study was observed in patients operated on with PI than in those operated on with CR. These differences were confirmed by effect-size analyses, in which higher values of Cohen’s d for ROE (3.48 vs. 3.07) and r for total SCHNOS (0.99 vs. 0.80), SCHNOS-O (0.85 vs. 0.62), and SCHNOS-C (0.97 vs. 0.93) indicated a stronger therapeutic effect in the PI group (Table 4).

**Figure 3:**
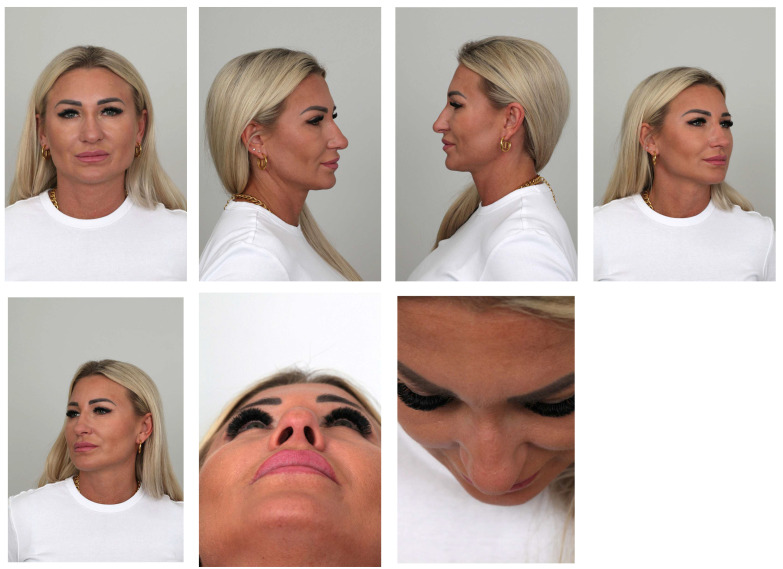
Preoperative photographs of a female patient undergoing rhinoseptoplasty with powered instrumentation (PI).

**Figure 4:**
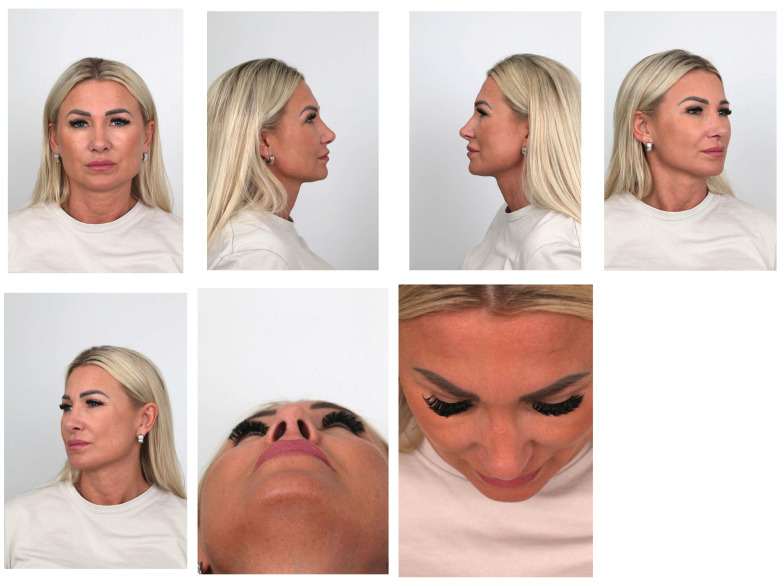
Six-month postoperative photographs of the same female patient after rhinoseptoplasty with powered instrumentation (PI).

These findings may suggest good effectiveness of PI in improving both aesthetic and functional aspects of rhinoseptoplasty. The observed differences may result from several factors related to the operative procedure itself and to the healing process. First, PI may provide better intraoperative control over the osteocartilaginous structures of the nose, enabling more precise osteotomy and more accurate restoration of nasal architecture. In addition, more predictable operative conditions may reduce soft-tissue injury, resulting in a less intense inflammatory response and less postoperative swelling. This may influence not only recovery speed but also the long-term stability of aesthetic outcomes. Consequently, these factors may contribute to more predictable and reproducible treatment results, both in subjective patient assessment and in objective clinical parameters. Nevertheless, these observations require further verification in larger studies with longer follow-up to better define the comparative performance of this technique. Figure 3 and Figure 4 show a patient before and after surgery performed with PI.

With regard to the most recent meta-analysis published in Aesthetic Surgery Journal, entitled “Piezoelectric Versus Conventional Rhinoplasty: A GRADE-assessed Systematic Review and Meta-analysis of Randomized Controlled Trials” by Armanfar et al. [26], the use of piezoelectric osteotomy may be associated with a significant reduction in early postoperative morbidity compared with conventional techniques. This is consistent with the findings presented in the present study. The meta-analysis showed reductions in postoperative edema, bruising, and pain, as well as a significantly lower risk of mucosal injury. At the same time, the authors indicated that osteotomy duration appears comparable between both techniques, although available data remain inconclusive and substantially heterogeneous. In the present study, operative time was not analyzed. Overall, the results of Armanfar et al. support the advantage of piezoelectric techniques in terms of soft-tissue safety and improved postoperative comfort [26].

After careful exposure of the operative field, bone contouring can be performed using conical burrs. The tapered shape of the burr allows free access to the narrow portion of the nasofacial groove, particularly in its distal segment, where the operative field may be limited. The purpose of maneuvers performed with powered instrumentation is to smooth the surface of the frontal process of the maxilla, create a sharper angle between the maxillary surface and the lateral nasal wall, and better define the nasofacial groove. At the same time, a smooth transition from the maxillary bone to the nasal bone can be ensured by eliminating undesired irregularities that often represent a major difficulty for the surgeon when using conventional instruments. Congenital or post-traumatic irregularities can be corrected with conical burrs in a more controlled manner than with conventional methods [8,27].

In our study, we observed that the use of conical burrs significantly reduced the need for intermediate osteotomies that would otherwise have been required during rhinoseptoplasty with conventional instruments to achieve a satisfactory aesthetic result. Intermediate osteotomies were performed in 10 of 62 patients (16.1%) in the CR group and in 2 of 44 patients (4.5%) in the PI group, in which conical burrs were used. In the case of a wide and convex nasal bone, conventional surgery often requires dividing the bone into two fragments to straighten it. With a conical burr, the bone can be flattened by thinning its middle segment, which allows a more predictable and aesthetic result with lower procedural invasiveness. In our practice, ball-shaped burrs are not used due to an observed tendency to produce surface irregularities on the bone. Such irregularities have not been encountered with the use of conical and cylindrical burrs, which provide more consistent bone surface modification [28]. Piezoelectric devices have been suggested to offer advantages in terms of precision, controlled cutting, and excellent visualization of the operative field, and they also allow for limited bone modification during osteotomy. The complementary roles of conical and cylindrical burrs used in combination with piezoelectric instrumentation are summarized in Table 5.

It should also be noted that osteoplasty performed with conical or cylindrical burrs may facilitate subsequent osteotomy, as prior bone thinning reduces resistance and may contribute to a more controlled cutting line, particularly when conventional osteotomes are used. The main advantage of the conical burr compared with the cylindrical burr is improved access to more distal areas of the operative field, especially around the radix and at the transition between the maxillary surface and the nasal pyramid. The narrower tip of the instrument facilitates more precise work in these anatomically challenging regions.

Conical and cylindrical burrs may also allow for subtle intraoperative modification of cartilage contours [9,28]. Although they are not intended as primary instruments for structural modification of cartilaginous frameworks, they can be useful for correcting minor irregularities of the nasal dorsum, which may help streamline the procedure and reduce operative time. This is particularly relevant after resection of the bony nasal vault during hump reduction, when cartilaginous components become more exposed in deeper layers. In such situations, these components are less responsive to piezoelectric instrumentation but can be effectively refined using burrs [17].

It should be emphasized that cartilage work requires significantly less force than bone manipulation. A gentle, brush-like contact with the tissue is recommended to minimize the risk of cartilage injury [35]. From a practical standpoint, cylindrical burrs may offer an advantage over conical burrs during cartilage contouring, as surgical access is usually favorable and the instrument’s geometry allows for more controlled shaping. The abrasive surface aligned with the axis of the handpiece facilitates the creation of a smooth and even nasal dorsum. It is generally suggested that the use of piezoelectric instruments may be associated with improved postoperative outcomes compared with conventional techniques. The findings of the present study are consistent with these observations (Table 4).

Another area that can be effectively addressed with powered instrumentation during rhinoseptoplasty is the osseous component of the nasal septum, including both distal structures (such as the anterior nasal spine) and proximal elements (such as the maxillary and palatine crests). In cases of marked bony deviation, conventional techniques, including osteotomes and partial resection of the deviated segment, are typically required. However, these approaches may be associated with complications such as bleeding from injured incisive vessels, transient numbness of the incisors due to osteotome use or bipolar electrocoagulation, and hematoma formation, which in rare cases may progress to septal abscess [17,30,36,37]. Management of such complications includes incision and drainage, nasal packing, and antibiotic therapy, while delayed treatment may result in septal loss and secondary saddle-nose deformity requiring further surgical correction.

In cases of less pronounced deviations involving the maxillary and palatine crests, cylindrical burrs may offer a useful alternative. They allow controlled thinning and reshaping of the bony contour, contributing to improved nasal airway patency while potentially reducing the risk of the complications associated with more invasive techniques. This approach is particularly useful in inverted T-shaped deformities, where bilateral bone thinning facilitates more harmonious septal realignment and may improve both functional and aesthetic outcomes.

In the distal septum, particularly around the anterior nasal spine, conical burrs are especially useful. In most cases, deviation in this region does not require complete or partial resection; gradual and controlled thinning under direct visualization is usually sufficient. It should be emphasized that the anterior nasal spine does not necessarily need to be perfectly midline. Partial reduction on the side of deviation, while preserving a noncentral position, allows application of the swinging-door technique, facilitating stabilization of the quadrangular cartilage on the contralateral side and improving both nasal symmetry and airway function [36,37].

In tension nose deformities, careful contouring of the anterior nasal spine using burrs enables controlled and safe correction. The use of an osteotome in this region carries a risk of uncontrolled fracture, which may compromise septal stability and lead to recurrent deviation, loss of tip and dorsal support, altered nasal projection and rotation, and secondary dorsal collapse, often necessitating revision surgery. The use of more delicate instruments such as conical burrs may reduce this risk and allow more predictable anatomical reshaping while maintaining postoperative stability.

The radix region has traditionally represented a technically demanding area in rhinoplasty due to limited access and constrained operative visibility. Conventional instruments such as rasps and osteotomes may be difficult to control in this region, often limiting the precision of bony reduction and potentially affecting aesthetic outcomes. The introduction of powered instrumentation has improved surgical access and control in this area [30,38]. In the present study, the observed improvement in ROE and cosmetic SCHNOS scores in the PI group may partly reflect enhanced contouring of the radix region compared with the CR group (Table 4; Figure 1, Figure 2, Figure 3 and Figure 4).

A key aspect of rhinoplasty is the reshaping of the bony nasal pyramid, where both congenital and post-traumatic asymmetries must be addressed. Achieving optimal symmetry remains a primary goal of aesthetic nasal surgery. Conventional osteotomy techniques may not always allow fully precise correction and can be associated with a risk of fragment instability and secondary postoperative asymmetries [18,39].

In a cadaveric study by Schlabe et al. entitled “A comparison of piezo surgery osteotomies with conventional internal osteotomies as performed by trainee surgeons,” piezoelectric instrumentation demonstrated improved precision and more controlled osteotomy lines compared with conventional techniques, even in a training setting [40]. These results suggest improved predictability with this technique and a potentially reduced risk of uncontrolled injury to bony structures [40]. Based on our experience, the use of burrs compared with conventional manual instruments such as rasps may provide greater control during bone contouring and reduce the likelihood of unintended irregularities. This may facilitate the achievement of a more symmetric and predictable outcome with a low complication profile. When comparing techniques, differences in surgical access and the mechanics of bone remodeling must also be considered, as these factors significantly influence both functional and aesthetic results [9].

Burr-assisted contouring can be performed through a limited surgical corridor created via an intercartilaginous incision within the nasal vestibule, allowing minor bony refinements even under local anesthesia. In clinical practice, this approach is particularly useful in revision cases presenting with subtle irregularities of the bony nasal pyramid [9].

By contrast, extensive osteoplasty with a burr requires adequate exposure. Attempting to work within a restricted field may increase the risk of soft-tissue injury and reduce control over the instrument tip, potentially resulting in either insufficient or excessive bone removal and a suboptimal aesthetic outcome [8,9,35].

During the procedures included in this study, no complications such as injury to the lacrimal system or persistent epiphora were observed in either group. No major intraoperative complications, including uncontrolled fractures or significant soft-tissue damage, occurred. Mild transient numbness of the nasal dorsum was reported in five patients (three in the CR group and two in the PI group) and resolved spontaneously without intervention, most likely due to temporary irritation of small sensory nerve branches during dissection. Postoperative bleeding was observed in two patients, one in each group. In both cases, bleeding was mild and was managed conservatively without surgical intervention. None of the patients required reoperation or urgent surgical revision during follow-up, supporting the safety of both CR and powered instrumentation techniques.

The duration of postoperative observation may appear as a limitation of the study; however, the authors would like to emphasize that the present study focused primarily on outcomes related to the bony vault, where postoperative stabilization and resolution of edema occur relatively early compared with other nasal subunits. In rhinoseptoplasty, swelling in the cartilaginous tip region may persist for up to 12 months or longer, whereas postoperative changes in the bony part of the nose typically become clinically apparent much earlier [2,6,13]. Therefore, the authors considered a 6-month follow-up sufficient to evaluate the early effects of the surgical techniques investigated in this study and their impact on patient-reported outcomes. Importantly, these early postoperative stages are also highly relevant from the patient’s perspective, as they substantially influence patient comfort, satisfaction, and psychological well-being during recovery, which was one of the aspects the authors aimed to assess. At the same time, the authors acknowledge that long-term evaluation remains important in rhinoplasty surgery. Further follow-up is currently ongoing, and extended assessment at 2 years postoperatively is planned to evaluate the stability of outcomes over time.

In summary, a potential advantage of powered instrumentation is the high precision of the procedure. Under visual control, it is possible not only to remove bony structures responsible for a nasal hump but also to model them, narrow the nasal pyramid, and lower the radix region. This may contribute to improved accuracy compared with conventional instruments, as suggested by the findings of the authors’ study. It is also worth emphasizing that these procedures can be performed after osteotomy, that is, on already mobilized bone fragments. This is practically impossible with manual rasps because they require considerable force and pressure, which may destabilize bony fragments, displace them, and create secondary nasal deformities. The use of burrs eliminates the need for high force and enables safe, controlled bone modeling. The lower mechanical load during work also substantially reduces the risk of injury to underlying cartilaginous structures.

## 5. Conclusions

In recent years, interest in aesthetic nasal surgery has increased markedly, accompanied by a growing need for predictable, functional, and aesthetically harmonious results. Contemporary rhinosurgery emphasizes not only the visual result but also preservation or improvement of nasal airway patency, which requires increasing precision and intraoperative control.

The use of powered instrumentation may increase the accuracy of nasal-bone modeling by enabling gradual and controlled preparation. This may minimize the risk of uncontrolled fractures and instability of bony fragments, which may occur with conventional instruments. As a result, the surgeon has a chance to achieve greater predictability of the final outcome and better control over nasal symmetry and proportions. This technique also facilitates a smooth transition between bony and cartilaginous structures, which is essential for the natural appearance of the nasal dorsum. The ability to precisely shape the nasofacial groove and radix region is particularly important because these regions play a key role in the aesthetic perception of the facial profile. These elements have traditionally been difficult to achieve with conventional instruments such as rasps and osteotomes. In addition, burrs allow more selective correction of bony asymmetries, often reducing the need for extensive osteotomies. This may decrease procedural invasiveness, reduce soft-tissue trauma, and potentially shorten recovery time while reducing postoperative symptoms such as swelling and bruising. It should also be emphasized that different types of burrs, particularly cylindrical and conical burrs, play complementary roles during surgery, enabling both preparation of larger bony surfaces and precise contouring of difficult-to-access areas. 

In summary, powered instrumentation represents an important addition to the contemporary rhinosurgeon’s armamentarium. Its use responds to the growing expectations of both patients and surgeons by aiming to improve precision, safety and predictability of outcomes. Further development of these technologies is likely to play a key role in the future of nasal surgery, particularly in the pursuit of natural, durable, and functionally optimal treatment results.

## Figures and Tables

**Table 1:** Comparison of ROE and SCHNOS scores before and after rhinoseptoplasty.

	Value (±SD)	*p*-Value (K-S)
ROE, preoperative	8.39 (±4.15)	>0.05
ROE, postoperative	19.92 (±3.08)	<0.05
SCHNOS, preoperative	33.49 (±10.06)	>0.05
SCHNOS-O (items 1–4), preoperative	10.95 (±5.95)	>0.05
SCHNOS-C (items 5–10), preoperative	22.54 (±6.60)	<0.05
SCHNOS, postoperative	7.02 (±7.28)	<0.05
SCHNOS-O (items 1–4), postoperative	2.99 (±2.83)	<0.05
SCHNOS-C (items 5–10), postoperative	4.03 (±6.03)	<0.05

**Table 2:** Age, sex, structure and questionnaire results in the PI subgroup.

	Value (±SD)	*p*-Value (K-S)
Patients	44	
Mean age	36.02 (±10.33)	
Women	30	
Men	14	
ROE, preoperative	8.09 (±3.92)	>0.05
ROE, postoperative	20.14 (±3.10)	>0.05
SCHNOS, preoperative	35.14 (±9.24)	>0.05
SCHNOS-O (1–4), preoperative	11.95 (±5.66)	>0.05
SCHNOS-C (5–10), preoperative	23.18 (±5.82)	>0.05
SCHNOS, postoperative	7.05 (±6.66)	<0.05
SCHNOS-O (1–4), postoperative	3.45 (±2.98)	>0.05
SCHNOS-C (5–10), postoperative	3.59 (±5.12)	<0.05

**Table 3:** Age, sex, structure and questionnaire results in the CR subgroup.

	Value (±SD)	*p*-Value (K-S)
Patients	62	
Mean age	32.55 (±9.18)	
Women	48	
Men	14	
ROE, preoperative	8.60 (±4.32)	>0.05
ROE, postoperative	19.76 (±3.08)	>0.05
SCHNOS, preoperative	32.32 (±10.53)	>0.05
SCHNOS-O (1–4), preoperative	10.24 (±6.09)	>0.05
SCHNOS-C (5–10), preoperative	22.08 (±7.11)	<0.05
SCHNOS, postoperative	7.00 (±7.75)	<0.05
SCHNOS-O (1–4), postoperative	2.66 (±2.69)	<0.05
SCHNOS-C (5–10), postoperative	4.34 (±6.62)	<0.05

**Table 4:** Comparison of preoperative and postoperative questionnaire scores in the subgroups.

	Preoperative	Postoperative	*p*	d	r	95% CI
ROE: PI, preoperative vs. postoperative	8.09 (±3.92)	20.14 (±3.10)	<0.0001	3.48		[−13.53, −10.57]
ROE: CR, preoperative vs. postoperative	8.60 (±4.32)	19.76 (±3.08)	<0.0001	3.07		[−12.48, −9.84]
SCHNOS: PI, preoperative vs. postoperative	35.14 (±9.24)	7.05 (±6.66)	<0.0001		0.99	[24.67, 31.51]
SCHNOS: CR, preoperative vs. postoperative	32.32 (±10.53)	7.00 (±7.75)	<0.0001		0.80	[22.03, 28.61]
SCHNOS-O: PI, preoperative vs. postoperative	11.95 (±5.66)	3.45 (±2.98)	<0.0001		0.85	[5.56, 11.44]
SCHNOS-O: CR, preoperative vs. postoperative	10.24 (±6.09)	2.66 (±2.69)	<0.0001		0.62	[5.9, 9.26]
SCHNOS-C: PI, preoperative vs. postoperative	23.18 (±5.82)	3.59 (±5.12)	<0.0001		0.97	[17.27, 21.91]
SCHNOS-C: CR, preoperative vs. postoperative	22.08 (±7.11)	4.34 (±6.62)	<0.0001		0.93	[15.3, 20.18]

**Table 5:** Comparison of preoperative and postoperative questionnaire scores in the subgroups [9,17,28,29,30,31,32,33,34].

Feature	Conical Burrs	Cylindrical Burrs
Shape	Tapered toward the tip	Constant diameter along the entire length
Clinical application	Hard-to-reach areas and precise corrections, e.g., radix and bony transitions	Broader surfaces, hump reduction, and smoothing of the nasal dorsum
Precision of cutting/contouring	High; enables selective, point-by-point modeling	Moderate; more global surface preparation
Access to difficult areas	Very good, particularly in the radix region and narrow spaces	Limited in deep and narrow areas
Bone-surface contouring	More precise and detailed	More uniform smoothing of larger surfaces
Risk of excessive bone removal	Lower in narrow areas because of better local control	Higher in restricted access; risk of depressions if used improperly
Stability of instrument guidance	Requires greater operator control	More stable on flat surfaces
Efficiency of tissue removal	Lower by volume, but more selective	Higher; faster preparation of larger areas
Visual control	Very good in a restricted field	Better in a wide operative field
Cartilage contouring	Possible for small focal irregularities, but requires delicate use	Useful for correction of minor cartilage irregularities
Soft-tissue safety	Requires great caution and minimal pressure	Requires great caution and minimal pressure
Reduction in need for intermediate osteotomies	Marked in selected convex or asymmetric bony areas	Significant after thinning of broader bony surfaces
Clinical usefulness	Optimal for precise local osteoplasty in narrow regions	Highly useful for osteoplasty and correction of surface irregularities
Complementary use	Complements piezoelectric osteotomy, particularly during contouring	Complements piezoelectric osteotomy, particularly during contouring

## Data Availability

The data presented in this study are available on request from the corresponding author. The data are not publicly available due to privacy.
